# Syndecan-1: From a Promising Novel Cardiac Biomarker to a Surrogate Early Predictor of Kidney and Liver Injury in Patients with Acute Heart Failure

**DOI:** 10.3390/life13040898

**Published:** 2023-03-28

**Authors:** Radu-Stefan Miftode, Irina-Iuliana Costache, Daniela Constantinescu, Ovidiu Mitu, Amalia-Stefana Timpau, Monica Hancianu, Daniela-Anicuta Leca, Ionela-Larisa Miftode, Raul-Alexandru Jigoranu, Alexandru-Florinel Oancea, Mihai Stefan Cristian Haba, Diandra Ioana Miftode, Ionela-Lacramioara Serban

**Affiliations:** 1 Department of Internal Medicine I (Cardiology), Faculty of Medicine, University of Medicine and Pharmacy “Gr. T. Popa”, 700115 Iasi, Romania; 2 Department of Immunology, Faculty of Medicine, University of Medicine and Pharmacy “Gr. T. Popa”, 700115 Iasi, Romania; 3 Department of Pharmacognosy, Faculty of Pharmacy, “Gr. T. Popa” University of Medicine and Pharmacy, 16 University Street, 700115 Iasi, Romania; 4 Department of Infectious Diseases, Faculty of Medicine, University of Medicine and Pharmacy “Gr. T. Popa”, 700115 Iasi, Romania; 5 Radiology Department, St. Spiridon Clinical Hospital, 700115 Iasi, Romania; 6 Department of Morpho-Functional Sciences II, University of Medicine and Pharmacy “Gr. T. Popa”, 700115 Iasi, Romania

**Keywords:** syndecan-1, acute heart failure, fibrosis, multi-marker

## Abstract

(1) Background: Acute heart failure (HF) represents a complex clinical syndrome burdened by increased mortality and a high rate of systemic complications. Although natriuretic peptides (e.g., NT-proBNP) currently represent the diagnostic and prognostic gold standard in acute HF, those molecules do not accurately reflect all the pathophysiological mechanisms involved in the progression of this pathology when determined independently. Therefore, the current paradigm tends to focus on a multi-marker approach for the risk stratification of patients with acute HF. Syndecan-1 is a less studied biomarker in cardiovascular diseases; its assessment in patients with acute HF being potentially able to reflect the myocardial pathological changes, such as fibrosis, inflammation, endothelial dysfunction or global wall stress. (2) Methods: We conducted a single center prospective study that enrolled 173 patients (120 patients admitted for acute HF, compared to 53 controls with stable chronic HF). A complete standardized clinical, echocardiography and laboratory evaluation was performed at admission, including serum samples for the determination of syndecan-1 by the enzyme-linked immunosorbent assay (ELISA) method. (3) Results: The serum concentration of syndecan-1 was significantly higher in patients with acute HF, compared to controls [121.4 (69.3–257.9) vs. 72.1 (41.4–135.8) ng/mL, *p* = 0.015]. Syndecan-1 was a significant predictor for the diagnosis of acute HF, expressed by an area under the curve (AUC) of 0.898, similar to NT-proBNP (AUC: 0.976) or cardiac troponin (AUC: 0.839). Moreover, syndecan-1 was independently associated with impaired kidney and liver function at admission, being also a predictor for early, subclinical organ dysfunction in patients with normal biological parameters at admission. When included in the multi-marker model, syndecan-1 levels influenced mortality more significantly than NT-proBNP or troponin. A multivariable regression including syndecan-1, NT-proBNP and troponin provided additional prognostic value compared to each independent biomarker. (4) Conclusions: Syndecan-1 can be considered a promising novel biomarker in acute HF, exhibiting adequate diagnostic and prognostic value. Additionally, syndecan-1 can be used as a surrogate biomarker for non-cardiac organ dysfunction, as its highs levels can accurately reflect early acute kidney and liver injury.

## 1. Introduction

Heart failure (HF) represents the “final destination” syndrome for most of the cardiovascular (CV) diseases; its outcome being further burdened by the commonly associated systemic complications. The increasing life expectancy in general population, doubled by modern therapeutic management that is reducing the short-term mortality (especially associated with acute ischemic events), determined the so-called HF pandemic, expressing its continuously growing prevalence worldwide [1,2].

Furthermore, acute HF represents the main cause of hospitalization in the elderly (>65 years old), emerging not only as a considerable source of mortality and morbidity but also as a continuously growing socioeconomic burden. Due to the rapidly unfavorable evolution of the patients presenting with suggestive symptoms for acute HF, it is mandatory to make a rapid and efficient diagnosis, doubled by an adequate risk assessment in order to improve their prognosis and minimize the burden associated with their hospitalization. In this context, it is of paramount importance to search for novel diagnostic tools and risk stratification instruments or, at least, to improve the currently existing ones [1,3].

According to the World Health Organization (WHO), biomarkers represent molecular parameters that are capable of providing objective and readily available information concerning certain physiological and pathological processes [4]. Natriuretic peptides (NPs) currently represent the cornerstone biomarker for the diagnosis of HF, both in chronic and acute settings. The latest 2021 HF guidelines of the European Society of Cardiology (ESC) recommend determining these molecules mostly for their negative predictive capacity, for this purpose providing certain cut-off values able to rule out an acute HF. However, when it comes to specificity, NPs display important limitations, as their serum level is influenced by numerous confounding factors, such as age, gender or comorbidities [5]. Considering this, it is acceptable to say that one should focus on the research of novel, less studied molecules, capable of a more accurate diagnosis and risk stratification of acute HF patients, either individually or as a part of a multi-marker approach.

Syndecan-1 is a transmembrane proteoglycan, being also a promising candidate for the range of HF biomarkers. Despite the literature data being scarce, there are some studies that evaluated the prognostic and diagnostic potential of this biomarker in patients with HF. Tromp et al., reported a direct correlation between serum syndecan-1 and NT-proBNP, also highlighting a strong relationship between increased syndecan-1 levels and multiple poor prognosis factors in HF, such as low blood pressure, reduced left ventricle ejection fraction (LVEF) and recurrent hospitalizations. When syndecan-1 was included in a composite multi-marker prognostic model, the same authors outlined that it increased the prediction precision, irrespective of the baseline LVEF [6]. The role of syndecan-1 in the pathogenesis of HF has been widely suggested, as it is associated with the cardiac fibrosis, atherogenesis, and neuroendocrine activation, especially related to the renin-angiotensin-aldosterone (RAA) system. In this regard, some experimental studies revealed that a low/non-detectable syndecan-1 is associated with a protective effect against Angiotensin II (Ang II)—induced cardiac dysfunction [7,8,9,10].

Moreover, syndecan-1 contributes to the pathogenesis of ischemic HF, playing an important role in the development of atherosclerosis. Wang et al., observed its overexpression in the macrophages found in atherosclerotic lesions, after the administration of Ang II, also remarking that the Ang II stimulates the shedding of the extracellular domain of syndecan-1. This process makes the ectodomain of syndecan-1 more available for different pro-inflammatory and pro-fibrotic circulating ligands, thus subsequently facilitating the progression of HF [11]. The list of the ligands, which are prone to interact with soluble syndecan-1, is variate, ranging from growth factors (vascular endothelial growth factor, transforming growth factors β1 and β2); cytokines (interleukins 2, 3, 4, 5, 7, 12, interferon, tumor necrosis factor α); or proteases (matrix metalloproteinase 7 and 9, cathepsin-G) [12].

Another aspect that supports the hypothesis that syndecan-1 is a potentially useful biomarker is related to endothelial dysfunction. Patients with HF are characterized by a markedly increased inflammatory environment, doubled by the exacerbated adrenergic reactivity that is further associated with an important endothelial dysfunction and glycocalyx injury. As syndecan-1 is an important component of the glycocalyx covering the endothelial cells, its serum levels tend to increase in HF patients due to the above-mentioned pathological phenomena. A recent study showed a statistically significant association between the inflammatory status and endothelial injury, syndecan-1 serum levels being linearly correlated with C-reactive protein (CRP). Other highlighted studies increased syndecan-1 levels amongst patients suffering from acute myocardial infarction (AMI), due to both adrenergic stimulation and ischemic-mediated endothelial dysfunction. Furthermore, in these studies the concentration of syndecan-1 increased proportionally to that of adrenaline, both molecules proving to be independent predictors for long-term mortality [8,13]. When it comes to short-term mortality risk, Fuernau et al., reported a significant association between high syndecan-1 levels and in-hospital death rate due to cardiogenic shock in AMI patients [14].

The relationship between kidney disease and heart failure is bidirectional, renal function impairment being also associated with endothelial dysfunction. A recent study showed that syndecan-1 may emerge as an early predictive biomarker for acute kidney injury (AKI) among patients admitted for acute HF. Noteworthy, its serum levels tend to increase in this clinical setting even before the actual onset of kidney dysfunction. Contrary to other biomarkers, which only become elevated once the glomerular filtration rate (GFR) decreases, syndecan-1 increases even in the presence of a continuous glycocalyx damage, as it is the “subclinical” AKI [7].

As previously mentioned, patients with HF are usually older and present a plethora of comorbidities, aspects that actually reflect the systemic continuum between the cardiac and non-cardiac pathologies. Another important characteristic of syndecan-1 is reflected by its major role in the liver diseases, as its serum concentrations tend to increase in those pathologies associated with liver fibrosis. Multiple studies have evaluated the role of this molecule as a potential biomarker for liver dysfunction in non-alcoholic liver disease, chronic viral hepatitis, toxic hepatitis or liver cirrhosis [8,12]. Contrary to HF, syndecan-1 has a protective effect in liver disease, its overexpression being associated with the inhibition of hepatic fibrosis. This can be explained by the fact that, by attaching to TGF-β1, syndecan-1 accelerates the clearance of this important promotor of hepatic fibrosis. Moreover, some studies showed that syndecan-1 may also reduce the serum levels of thrombospodin-1, a powerful activator of TGF-β1, thus enhancing its hepatoprotective effects. Of course, despite its overall beneficial effects, the presence of persistently increased levels of syndecan-1 generally reflects the activity of the liver aggression or a more rapid progression of a chronic, previously stable liver disease [8,15].

**Figure 1:**
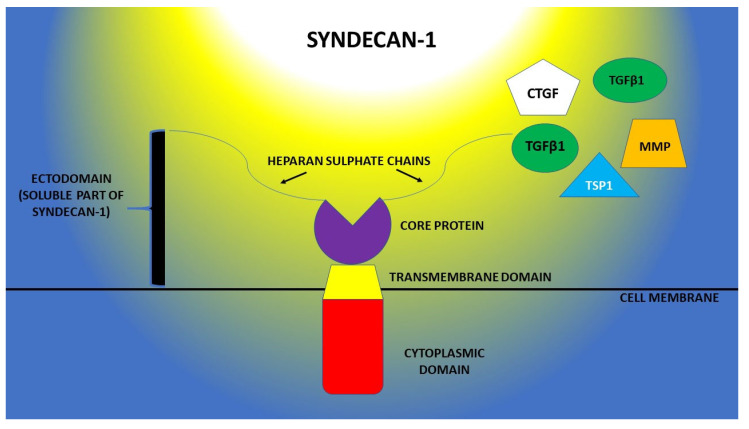
Structural aspects of syndecan-1 and the ligand molecules of its ectodomain (TGFβ1—β-type transforming growth factor; MMP—matrix metalloproteinases; CTGF—connective tissue growth factor; TSP1—thrombospondin-1).

Focusing on its structural characteristics, syndecan-1 is consisting of multiple glycosaminoglycan (GAG) chains that are attached to a core-protein, this molecular architecture being responsible for the specific biologic effects (Figure 1). The core-protein has a molecular mass of 20–40 kDa, and it is composed of a cytoplasmic C-terminal domain, a transmembrane one and the last one which is extracellular and represents the attaching site for GAG [8,15]. Another aspect worth mentioning is that syndecan-1 is semi-ubiquitous, being expressed in multiple tissues and organs, and capable of binding different types of GAG, such as heparan-sulfate (HS) and chondroitin-sulfate (CS), which can further serve as binding sites for other different ligands. This makes syndecan-1 a very versatile molecule, which is involved in an impressive number of intracellular signaling pathways [16].

In this study we aimed to assess the diagnostic and prognostic capacity of syndecan-1 in patients admitted for acute HF. Furthermore, we evaluated the various correlations between the serum levels of this biomarker and other comorbidities (kidney and liver dysfunction) that are associated with a negative prognostic in patients with HF.

## 2. Materials and Methods

### 2.1. Study Design, Patients and Investigations

We conducted a prospective case-control study that included 120 patients who presented to St. Spiridon Emergency County Hospital (Iasi) between January 2021 and June 2021 for sudden-onset (or rapidly progressive) dyspnea. They were subsequently admitted to the Cardiology Clinic for acute HF, irrespective of its clinical phenotype, as described by the latest ESC guidelines (e.g., acute decompensated HF, acute pulmonary edema, cardiogenic shock, isolated right ventricular failure). Chronic HF was clinically established according to the Framingham criteria, two major (paroxysmal nocturnal dyspnea, orthopnea, jugular veins distention, third heart sound, cardiothoracic ratio >0.5, pulmonary edema or pulmonary crackles) or one major + two minor criteria (peripheral edema, nocturnal dyspnea, exertion dyspnea, hepatomegaly, pleural effusion and heart rate ≧ 100/min) being mandatory for the clinical diagnosis. An echocardiography exam was performed at admission in every patient, using a GE VIVID™ V7 (General Electric, Boston, CA, USA) ultrasound device, which assessed the general morphofunctional cardiac characteristics, such as chamber dimension, systolic and diastolic function or pulmonary hypertension, also excluding other secondary causes for the acute decompensation (e.g., cardiac tamponade, acute valvular heart disease).

The control group consisted of 53 patients with previously diagnosed stable HF who presented for regular ambulatory follow-up visits and had no admissions due to HF in the past year.

We excluded the patients with acute HF who refused to participate in the study or those who were diagnosed with a severe or end-stage pathology (e.g., terminal cancers, dialysis or kidney transplant list, Child C liver cirrhosis, severe sepsis, etc.). Another exclusion criteria was a NT-proBNP value below the ESC recommended 300 pg/mL cut-off or the presence of documented neuropsychiatric disorders. Patients who could not be properly examined due to certain individual particularities (e.g., important obesity or poor echocardiographic window) were also excluded from the study.

All the included patients underwent a complete physical examination, detailed anamnesis and medical history assessment, including comorbidities, relevant laboratory parameters and sociodemographic information. A 12-lead ECG was also recorded early after admission, followed by a complete standard laboratory test panel (hemogram, kidney and liver function, lipid profile) for the entire study and control cohort. Classical biomarkers, such as NT-proBNP and high-sensitive cardiac troponin I (hs-TnI) were assessed during the first hour after admission in the Cardiology Clinic.

Acute kidney injury (AKI) was defined according to RIFLE criteria, starting with a 1.5-fold increase in serum creatinine, doubled by a 25% decrease in glomerular filtration rate in a patient, with an urinary output lower than 0.5 mL/kg/h for at least 6 h [17]. For AKI diagnosed “early after admission” we considered the changes in kidney function occurring in the first 24–48 h of hospitalization, including the changes in serum creatinine or urine output, according to the aforementioned RIFLE criteria. Liver cytolysis was defined according to the reference values of the hospital laboratory, as any value above the upper normal limit for gender-adjusted aspartate aminotransferase (45 U/L) or alanine aminotransferase (48 U/L). The acute liver injury assessment was based on a two- to three- times elevation of transaminases (as a marker of liver damage) associated with impaired liver function, i.e., jaundice or coagulopathy (INR > 1.5). Alcohol abuse was defined as regular consumption of >2 standard drinks for men and >1 for women.

A 2 mL sample of venous blood was collected at admission and centrifuged at 2000 rpm for 20 min. The obtained serum samples were adequately stored (−80 °C) until the integrative biomarker analysis of the entire cohort. Syndecan-1′s quantification was performed using ELISA CD 138 ab46506 kits (Abcam, Waltham, MA, USA), with a detection rate of 8–256 ng/mL. The absorption readings were made at 450 nm using an Infinite 200 PRO M Plex Microplate Reader (Tecan, Grödig, Austria). Initially, the samples were diluted and combined with the provided antibody cocktails, according to the protocol indicated by the manufacturer. The quantitative assessment of serum syndecan-1 from each patient was performed with a Magellan Pro v.7.4 software (Tecan, Grödig, Austria), by intersecting the corresponding reading of each sample on the standard curve.

The primary endpoint was to assess the variation and diagnostic value of syndecan-1 in patients with acute HF, compared to the control group with chronic HF. Secondary endpoints consisted in evaluating the role of an increased syndecan-1 as a predictor of systemic dysfunction in the setting of acute HF.

### 2.2. Statistical Analysis

We performed the statistical analysis using the SPSS v.26 software (IBM, Armonk, NY, USA). In all the analyses, a *p*-value < 0.05 represented the threshold for statistical significance.

We used the Kolmogorov-Smirnov test to assess the normal distribution of continuous variables in the included patients. Mean ± standard deviation (STD) was used to express normally distributed variables, while the not-normally distributed data were expressed as medians with interquartile ranges (IQRs: 25–75%). Frequencies and percentages were used to express descriptive data for categorical variables, while *t*-test and Whitney-U test served for assessing the differences between variables within the population’s various subgroups. The correlations between the different parameters were assessed by evaluating Pearson’s (for continuous variables) or Spearman’s (for categorical variables) *r* coefficients, as appropriate.

In order to determine the diagnostic performance of the included biomarkers, we compared the areas under the curve (AUC) that resulted from receiver operating characteristic (ROC) analysis. The same analysis was useful in determining relevant diagnostic and prognostic cut-off values for syndecan-1.

### 2.3. Ethics

We conducted this study according to the ethical principles mentioned in the Declaration of Helsinki (revised in 2013). At admission, all patients signed a standard written informed consent in order to participate in the study. The research protocol was validated by the local Ethics Committees of both the University of Medicine and Pharmacy “Gr.T.Popa” (no. 9537/2020, no. 280/2023), and of the St. Spiridon Emergency Clinical Hospital (no.41/2020).

## 3. Results

### 3.1. Baseline Characteristics

We enrolled 173 patients, further divided into two subgroups: 120 patients admitted in emergency for acute HF, compared to 53 controls with chronic, stable HF, who were evaluated in ambulatory. The overall 30-day mortality rate was 15%, with all 26 fatalities occurring in patients with acute HF, of which 21 (12.1%) deceased during hospitalization.

In Table 1, we summarized the demographic, clinical and biological characteristics of the patients who participated in the study. We found a significantly higher prevalence of important cardiovascular risk factors among patients with acute HF, such as age, obesity, alcohol abuse and a low level of HDL-cholesterol, compared to the baseline characteristics of the patients from the control group. However, regarding other traditional cardiovascular risk factors (gender, smoking status, hypertension or diabetes mellitus), there were no noteworthy differences between the two groups.

Apart from supportive therapy and inotropic agents (exclusively administered in hospitalized patients), the use of loop diuretics (i.e., furosemide) and mineralocorticoid receptor antagonists (i.e., spironolactone) was significantly more common among patients with acute HF (*p* < 0.01). However, beta-blockers and renin–angiotensin inhibitors (either angiotensin converting enzyme inhibitors or angiotensin receptor blockers) have been predominantly prescribed in controls as part of the standard therapeutic management of the clinically stable chronic HF.

### 3.2. Profile of Syndecan-1 in Acute HF

Since syndecan-1 is a relatively less studied biomarker in HF, the first step was to assess its serum concentration in the two subgroups. We observed that patients with acute HF had a significantly higher concentration of syndecan-1, compared to their counterparts with chronic HF (Table 2).

**Figure 2:**
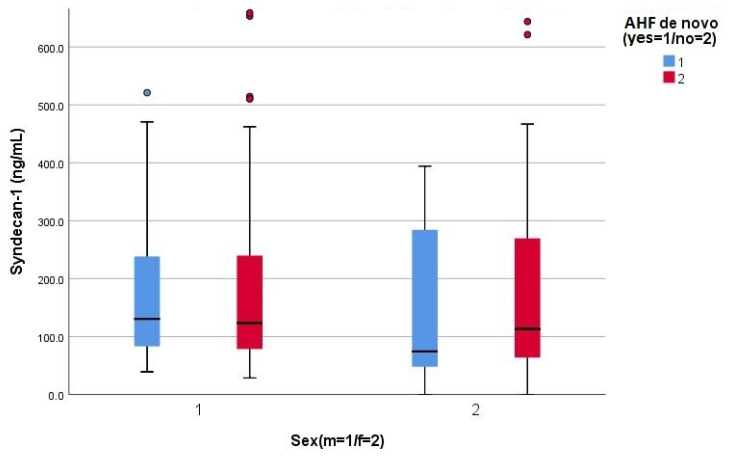
Clustered Boxplot of syndecan-1, by gender (male = 1, female = 2) and by de novo acute HF (1 = yes, 2 = no).

In the context of assessing a relatively novel biomarker, we hypothesized that the gender and onset type of acute HF (de novo, or a decompensation of a pre-existing chronic HF) may influence the serum concentration of syndecan-1; the results refuted this hypothesis for both gender (175.2 vs. 163.1 ng/mL, *p* = 0.675) and the onset type of HF (197.2 vs. 192.7 ng/mL, *p* = 0.878) (Figure 2).

### 3.3. Diagnostic Performance of Syndecan-1 in Acute HF

**Figure 3:**
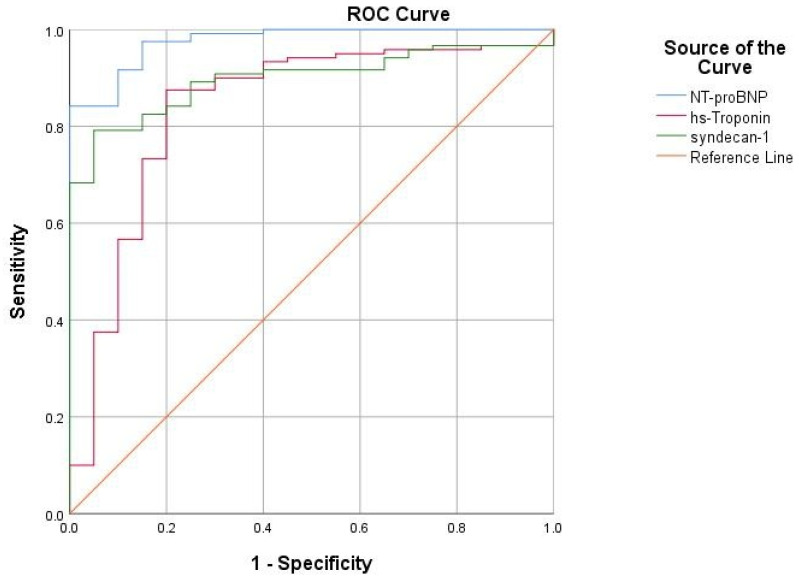
The ROC curve expressing the association between cardiac biomarkers and the diagnosis of acute HF.

The assessment of diagnostic performance is essential for biomarkers used in emergency settings, especially when one suspects acute HF in a patient with suggestive symptoms. All three assessed biomarkers were significant predictors for acute HF (Table 3); ROC analysis exhibited a consistent predictive value for syndecan-1, mirrored by its AUC of 0.898, showing a statistically significant performance (*p* < 0.05) in predicting acute HF, only slightly inferior to the gold-standard NTproBNP (AUC = 0.976) but substantially higher than that of hs-troponin (AUC = 0.838) (Figure 3).

From the ROC curve, we extracted a relevant diagnostic cut-off value of 73 ng/mL, corresponding to the established Youden’s index, which reflects the maximum sum of sensitivity (74.2%) and specificity (50.9%) related to syndecan-1. Regarding the high-risk threshold, at the limit of statistical significance [OR 2.57 (95% CI 0.97–7.01), *p* = 0.057], we identified that the concentration of 88.5 ng/mL is 60.8% sensitive and 41.5% specific in predicting increased mortality. This value is below the median value of syndecan-1 from our study group, which is 121 ng/mL.

### 3.4. Syndecan-1: Correlations with Echocardiographic Parameters in Acute HF

We found no significant correlation between serum syndecan-1 and the left ventricular end-diastolic diameter (LVEDD) or systolic function of the LV (LVEF). Although we observed a linear increase in syndecan-1 levels with increasing PAPs, it did not reach the statistical significance threshold. However, an elevated serum syndecan-1 was significantly associated with increased LV filling pressures, expressed as E/e’ (Table 4).

### 3.5. Syndecan-1: Potential Prognostic Role in Acute HF?

As for the prognostic ability or indication of severity of disease expressed in the need of inotrope drugs and ventilatory support, syndecan-1 was significantly correlated with the need for positive inotropic support and with the use of non-invasive ventilation mode. The fatality rate (both during hospitalization and 30 days after discharge) was not substantially associated with its serum levels. We observed that the increased concentrations of all biomarkers were directly correlated with mortality but only in the case of NT-proBNP in a significant manner (Table 5).

Consequently, we aimed to assess the potential complementary or additive value of syndecan-1 and NT-proBNP in predicting fatal events. For this purpose, we conceived a simple, easy-to-use, reproducible tool by comparing the overall mortality recorded in patients with acute HF according to the median serum levels of the biomarkers obtained in our study cohort. The results outlined the synergistic effect between syndecan-1 and NT-proBNP in predicting risk of death, the highest mortality rate occurring in patients presenting increased serum concentrations of both biomarkers (50% vs. 11.5%, *p* < 0.01). Not only did the mortality risk follow the rising concentration gradient, but it was more dependent on the particular increase of syndecan-1, compared to the one of NT-proBNP. Basically, the estimated risk was significantly higher in patients in whom syndecan-1 was above the median value, regardless of the intrinsic median concentration of NT-proBNP. A very similar pattern was also observed for the model including hs-troponin, the mortality being influenced exclusively by the variation of the syndecan-1 (Table 6).

### 3.6. Syndecan-1 and the Risk Factors for HF

In Table 7, we compared the influence exerted on syndecan-1 by certain anthropometric, hemodynamic, biochemical or anamnestic parameters. We also sought to assess the potential bi-directional relationship with those aspects that influence the pathogenesis of HF via different pathways, such as endothelial dysfunction, oxidative stress, inflammation or fibrosis. At admission, we observed significantly higher levels of syndecan-1 in patients with hepatic cytolysis, tachycardia (>100 beats/minute) or kidney dysfunction. Although mean syndecan-1 levels were higher among patients who were smokers or those with a history of chronic ethanol consumption, with pre-existing liver or kidney pathology, the difference was not statistically significant. We also noted that low blood pressure (systolic BP < 90 mmHg) was relevantly associated with increased levels of syndecan-1, while an impaired systolic function (LVEF < 40%) did not considerably influence the concentration of biomarker.

### 3.7. Syndecan-1: Surrogate Marker of Liver and Kidney Injury

**Figure 4:**
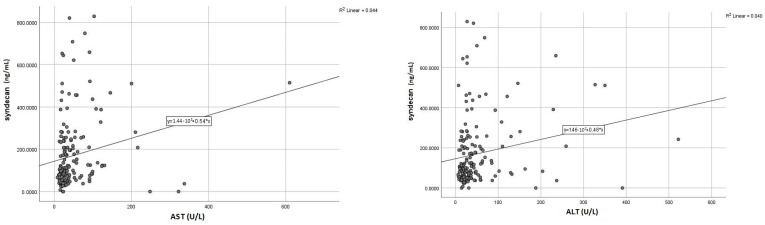
Positive direct correlations between syndecan-1 level and liver enzymes (AST—**left**; ALT—**right**).

**Figure 5:**
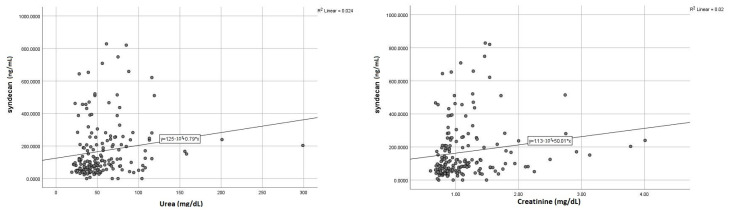
Significant direct correlations of syndecan-1 (serum urea—**left**; creatinine **right**).

**Figure 6:**
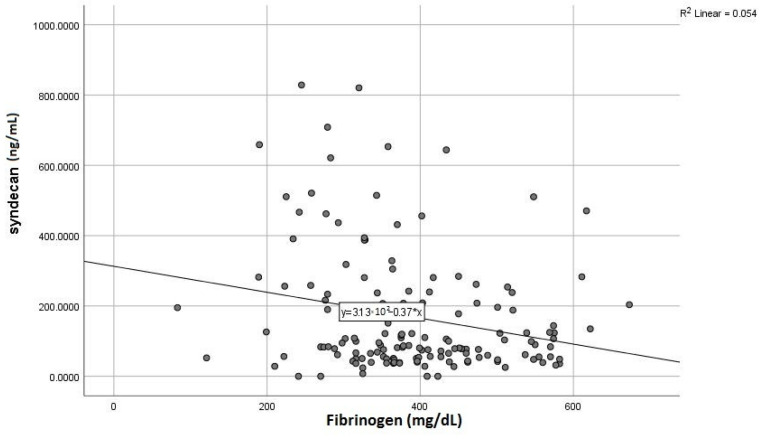
Inverse correlation between syndecan-1 level and serum fibrinogen.

Given those initial results, we further sought to assess the exact correlations (r) between syndecan-1 and specific parameters of the liver and kidney dysfunction, aiming to estimate its potential role as a dual biomarker. Accordingly, we found positive and meaningful correlations between elevated syndecan-1 and liver transaminases (Figure 4), a similar pattern being noticed for the correlation with serum urea and creatinine (Figure 5). Concerning the markers expressing the liver synthesis capacity, we noted a significant negative association with fibrinogen (Figure 6), as opposed to total serum proteins or INR, which did not display a noteworthy correlation.

Moreover, increased syndecan-1 may be used as an early predictor of organ dysfunction, as its elevated serum levels at admission were significantly and directly correlated with increased markers of liver and kidney injury occurring during hospitalization or before discharge, compared to initially normal baseline values (Table 8).

### 3.8. Syndecan-1 and Metabolic Profile

The cardiovascular risk profile includes a mandatory lipid panel assessment in each patient. We considered it appropriate to correlate these metabolic data with serum syndecan-1 levels, thus observing only a substantial negative correlation with HDL-cholesterol levels but not with total cholesterol, serum triglycerides or LDL-cholesterol (Table 9).

## 4. Discussion

Despite the growing awareness among clinicians, researchers and even patients, HF remains one of the main sources of morbidity, mortality and significant healthcare costs in both developing and developed countries [18]. In the context of a globally rising incidence and prevalence of HF [19], doubled by a polymorphic clinical presentation that leads to high rates of misdiagnosis, the need for rapid and accurate diagnostic tools is becoming of utmost importance. Therefore, cardiac biomarkers have lately represented a fertile research area concerning the diagnostic approach of HF, particularly in acute HF. Even if there is a plethora of new HF biomarkers [20], in this study we focused on syndecan-1, a molecule apparently of less interest in cardiovascular pathology. Despite there being some data in the literature [6,21] supporting the use of syndecan-1 as a marker of glycocalyx injury and endothelial dysfunction, the results on its role in acute HF are extremely scarce.

Starting from these benchmarks, we initially highlighted the significantly higher concentration of syndecan-1 in patients with acute HF, compared to the control group with chronic HF, a pattern similar to that of one of the few studies that evaluated syndecan-1 in acute HF [7].

Secondly, regarding the possible discriminative ability of syndecan-1 in predicting acute HF in a heterogenous population, in our study we observed a substantial diagnostic performance exhibited by this novel biomarker, mirrored by its AUC of 0.898, a value similar to that of established biomarkers, such as NT-proBNP and hs-troponin. Moreover, from the ROC curve, we plotted a diagnostic cut-off value of 73 ng/mL, which is lower than the references of 120 or 125 ng/mL, previously reported by Neves [7] and Wernli [22], respectively. However, contrary to these studies, we did not observe a significant correlation between syndecan-1 levels and overall mortality (r = 0.031, *p* = 0.709).

Multiple data are invoking the bidirectional relationship between syndecan-1 and kidney dysfunction [7,8]; this hypothesis is gaining ground for two reasons: the importance of glycocalyx injury on the pathophysiology of cardiorenal syndrome is well-known, the second reason is the possibility of using syndecan-1 as a biomarker for early detection of acute kidney injury (AKI). Our results confirmed this paradigm as we observed that syndecan-1 has a significant direct correlation with both baseline creatinine (r = 0.158, *p* = 0.031) and serum urea (r = 0.156, *p* = 0.041). However, the currently available data argue for this association only in patients who developed AKI just prior to admission, with no significant correlations observed in patients with previously diagnosed stable chronic kidney disease [7]. In our research, we found that increased syndecan-1 was associated not only with AKI diagnosed early after admission (within the first 24–48 h) but also with AKI developed later during hospitalization, as its levels were significantly higher even in patients with initially normal kidney function.

Under these circumstances, syndecan-1 may also emerge as an adequate biomarker for the early assessment of AKI, especially in patients with acute HF. These data may support the current evidence, which are suggesting that syndecan-1 levels are not influenced by the intrinsic decreased creatinine clearance but rather by the continued damage to the renal endothelial glycocalyx [23]. In patients with acute HF, this theory is allegedly based on a preexisting increased serum level of syndecan-1 at admission, before the actual onset of AKI and the subsequent decrease in creatinine clearance. Several authors have even raised the possibility of dosing syndecan-1 in patients with HF and AKI instead of the well-established kidney injury molecule-1 (KIM-1) and neutrophil gelatinase-associated lipocalin (N-GAL). Those studies basically suggested that early prevention of glycocalyx damage might be the appropriate therapeutic management to prevent the progression to AKI, which is burdened by a poor prognosis in patients with acute HF [7,24,25]. It should be mentioned, however, that only the urinary dosage of syndecan-1 would be an adequate tool to resolve this controversy.

Another area of promising research regarding syndecan-1 is its involvement in fibrotic processes, both myocardial and hepatic. We previously highlighted the use of syndecan-1 as a reliable marker of liver fibrosis [8], given the involvement of its extra membrane domain in the synthesis of MMPs, particularly MMP-14. The importance of MMPs resides in the fact that these molecules are directly involved in the degradation of ECM (extracellular matrix), thus preventing the progression of liver fibrosis. Moreover, there are additional alternative pathways to combat fibrogenesis: it has been shown that the circulating syndecan-1 isoform can bind thrombospondin, an activator of TGF-β1, and at the same time, it can accelerate its clearance [26]. As a result, syndecan-1 levels are elevated in most pathologies evolving with liver fibrosis, such as non-alcoholic steatohepatitis, liver cirrhosis or hepatocellular carcinoma. Our study further confirms these findings as we found strong positive associations between syndecan-1 and hepatic transaminases and inverse correlations with serum fibrinogen as a direct marker of liver synthesis function. In addition, patients with hepatic cytolysis at admission had significantly higher serum syndecan-1 values than those with transaminases within normal limits. In the same line with the AKI, we observed that increased syndecan-1 at admission was a strong predictor for liver cytolysis in patients with acute HF and initially normal baseline transaminases. Additionally, we noted that, although the biomarker presented higher levels in patients with a previously diagnosed liver pathology, the differences were not significant. One possible explanation is that the presumably protective effect exerted by syndecan-1 is only of limited duration (60–120 days), after which a steady decrease of its circulating levels is occurring, thus suggesting not only an impaired synthesis but also a limitation of its positive effects, as shown in a 2019 study by Regős et al. [26].

In acute HF, glycocalyx degradation may also be induced by neurohormonal hyperactivation of both the sympathetic nervous system and renin-angiotensin-aldosterone system, as an initial compensatory response to the pathophysiological changes (decreased periphery perfusion and increased myocardial wall stress) associated with cardiac dysfunction. Consequently, we observed that syndecan-1 was substantially increased in cases with tachycardia at admission (HR > 100/minute), a condition commonly met in patients with HF. Syndecan-1 was also directly correlated with elevated concentrations of the gold-standard NT-proBNP, thus further reflecting the indirect expression of increased myocardial wall stress and neurohormonal activation. This aspect may also be explained by the fact that natriuretic peptides increase endothelial permeability and, implicitly, the release of endothelium-attached molecules, such as syndecan-1 [27].

Inflammatory status is another aggravating factor of endothelial dysfunction, and it is routinely observed in patients with HF [13]. We found significantly higher syndecan-1 concentrations in patients with confirmed infectious pathology (including COVID-19), but when comparing strictly to the relevant C-reactive protein (CRP), the association was no longer significant (*p* = 0.234). Similar results were reported by Tromp et al., who found no significant correlations between CRP and syndecan-1 (*p* = 0.635) [28]. There are several studies incriminating the influence of inflammation (expressed as CRP) in increasing cardiac biomarkers [29,30,31]; thus, this atypical pattern of syndecan-1 compared to CRP is even more interesting. A possible explanation resides in syndecan-1’s capacity of shedding its extramembrane domains, a process that is highly involved in the resolution of inflammation. Specifically, shedding syndecan-1 binds to a plethora of inflammatory chemokines, such as chemokine ligand (CCL)-7, CCL-11 and CCL-17, thus enhancing their clearance and minimizing the subsequent recruitment of leukocytes or other inflammatory cells [32].

Nowadays, the link between dyslipidemia and endothelial dysfunction is widely accepted; the latter is the key step in the initiation and subsequent progression of atherosclerosis. One study previously reported the possible role of syndecan-1 in atherogenesis [33]; other authors even outlined the significant increase of serum syndecan-1 in patients with acute coronary syndromes. It was suggested that endothelial glycocalyx injury increases the vulnerability of atherosclerotic plaque, thus predisposing to acute ischemic events [34]. Following the statistical analysis, we observed a significant negative correlation between syndecan-1 and HDL-cholesterol, virtually confirming the role of HDL deficiency in endothelial dysfunction. The protective role associated with a normal HDL is mainly attributed to its pleiotropic effects [35], such as antioxidative, antiapoptotic, anti-inflammatory, antithrombotic or antiproteolytic activity on endothelial cells, thus preventing glycocalyx injury and the subsequent syndecan-1 release. Supplementary, syndecan-1 is overexpressed in atheromatous lesions of the aorta, such as fatty streaks and fibrolipid lesions, but also in intimal smooth muscle cells, thus further stimulating the formation of foam cells and making the atheroma plaque even more vulnerable [8,36]. The expression of syndecan-1 even in early lesions may render reasonable its inclusion in some currently available risk scores or even as a marker of subclinical atherosclerosis [37].

Two results caught our attention: the significantly higher values of syndecan-1 in hypotensive (BP < 90 mmHg) and non-obese patients (BMI < 30 kg/m^2^), respectively. These apparently paradoxical data can be interpreted in the key of HF severity. In the terminal stages of this pathology, patients have impaired cardiac output and low blood pressure (possibly requiring inotropes and vasopressors), but also cachexia-through nutrient absorption and metabolism deficit, both of which are classical predictors of poor prognosis [38,39]. Furthermore, Frydland et al., demonstrated that hemodynamically unstable patients with hypotension or cardiogenic shock have significantly higher syndecan-1 levels compared to hemodynamically stable ones who do not require inotropic or vasopressor support [40]. We report similar results, with a significant positive correlation between syndecan-1 concentration and the need for inotropic support (r = 0.206, *p* = 0.024).

Echocardiographic assessment in a patient with acute HF is mandatory and provides important prognostic information. We noted that syndecan-1 levels are not influenced by ventricular systolic function, expressing similar values irrespective of the LVEF (*p* = 0.663), in line with the results reported by Neves et al. (*p* = 0.520) [7]. This aspect might suggest the complex pathways expressed by this biomarker in the pathophysiology of subclinical HF, long before the apparition of wall stress due to pressure or volume overload. However, one study highlighted major differences (*p* = 0.036) between groups of patients with acute HF divided according to LVEF, elevated syndecan-1 levels being associated with significantly impaired systolic function [6].

To summarize, syndecan-1′s solid diagnostic performance in acute HF, similar to the one of classic biomarkers (NT-proBNP, hs-troponin), might open a new perspective concerning the use of novel biomarkers. The interchangeable use of syndecan-1 with the validated biomarkers can be feasible in certain situations, such as acute HF with preserved LVEF, given that syndecan-1 varies independently of this parameter. Another practical situation might refer to a dynamic assessment of syndecan-1 in order to evaluate the response to antifibrotic therapies in HF, such as RAAS inhibitors, thus potentially guiding the therapeutic management. The syndecan-1′s potential use in clinical practice is further based on its preserved ability as an early marker of fibrosis and a direct indicator of endothelial dysfunction in asymptomatic patients, with incipient and subclinical HF. In patients with already diagnosed HF, the potential role as a surrogate biomarker is reasonably supported by its capacity of detecting early, subclinical, liver and kidney injury, even before the alterations of the specific markers of organ dysfunction, such as serum creatinine or liver transaminases, as the negative prognostic role of these comorbidities in HF and vice versa is well-known.

### Study Limitations

The major limitations of the study consisted of the single-center design of the research, with the enrollment of a rather limited number of patients. The COVID-19 pandemic further hampered the total numbers of the cases, due to strict admission protocols and decreased addressability among the patients. Another drawback was represented by the scarcity of the syndecan-1 kits, as only one test was available for each patient, at admission. A further dynamic assessment of the biomarker at discharge and at the follow-up visits would certainly add prognostic value in HF.

## 5. Conclusions

To the best of our knowledge, this study is the first to evaluate the use of syndecan-1 in patients with acute HF from Eastern Europe, with those promising preliminary results turning the spotlight on this relatively novel biomarker.

We focused our research on depicting the potential use of syndecan-1 in clinical practice as a diagnostic and prognostic biomarker in patients presenting with phenomena suggestive of acute HF, especially from the perspective of systemic comorbidities and endothelial dysfunction. Syndecan-1 levels are significantly higher amongst patients with acute HF, compared to controls with stable HF. Moreover, its diagnostic potential is similar to the one of NT-proBNP or hs-troponin, making it a promising surrogate biomarker. Syndecan-1 also exhibited a superior prognostic role when included in a multi-marker test, the mortality rate being more dependent on the variation of syndecan-1 concentration than on that of classical biomarkers.

Last, but not least, we emphasize that syndecan-1 is significantly associated with elevated markers of kidney and liver injury, either at admission or during hospitalization.

## Figures and Tables

**Table 1:** Baseline characteristics of patients with acute HF and control group.

Characteristics		Acute Heart Failure (No = 120)	Control Group (No = 53)	*p*-Value
		Mean ± STD	Mean ± STD	
Age (years)		66.4 ± 15.3	64 ± 11.9	0.526
In-hospital mortality rate: No, (%)		21 (12.1%)	0 (0%)	<0.001
**Gender**	Men	71 (59.20%)	33 (62.30%)	0.438
	Women	49 (40.80%)	20 (37.70%)	
Smoking: No, (%)		48 (40%)	19 (35.8%)	0.605
Alcohol abuse: No, (%)		75 (62.5%)	22 (41.5%)	0.012
Arterial hypertension: No, (%)		60 (50%)	34 (64.2%)	0.085
Ischemic heart disease: No, (%)		59 (49.2%)	17 (32%)	0.037
Diabetes mellitus No, (%)		22 (18.3%)	7 (13.2%)	0.406
BMI > 30 kg/m^2^ N, (%)		42 (35%)	7 (13.2%)	0.003
LVEF		33.8 ± 13.9%	52.2 ± 15.7	0.017
Anemia: No, (%)		35 (29.2%)	12(22.7%)	0.377
Loop diuretics		102 (85%)	17 (32.1%)	<0.001
MRA		83 (69.2%)	9 (17%)	<0.001
Beta-blockers		99 (82.5%)	49 (92.5%)	0.087
RAS inhibitor		77 (64.2%)	44 (83.1%)	0.012

BMI—body mass index; LVEF—left ventricle ejection fraction; MRA—mineralocorticoid receptor antagonist; RAS—renin-angiotensin-system.

**Table 2:** Concentration of syndecan-1 and classical biomarkers in patients with acute and chronic HF.

Biomarker	Patient Group	No.	Median (IQR:25–75)	*p*-Value
Syndecan-1 (ng/mL)	Acute HF	120	121.4 (69.3–257.9)	0.015
	Control group	53	72.1 (41.4–135.8)	
NT-proBNP (pg/mL)	Acute HF	120	5440 (2812–12791)	<0.01
	Control group	53	107.8 (41.3–325.2)	
High-sensitive Troponin (ng/L)	Acute HF	120	40.1 (12.4–179.5)	<0.01
	Control group	53	2.2 (1.1–5.4)	

**Table 3:** Detailed AUC for the specified biomarkers.

Area under the Curve				
Test Result Variable(s)	Area	*p*	Asymptotic 95% Confidence Interval	
			Lower Bound	Upper Bound
NT-proBNP	0.976	<0.0001	0.952	1.000
hs-troponin	0.839	<0.0001	0.733	0.944
Syndecan-1	0.898	<0.0001	0.845	0.951

**Table 4:** Correlations between syndecan-1 and relevant echocardiographic parameters in acute HF.

Echocardiographic Parameter	Syndecan-1	
	*p*	r
LVEF	0.663	−0.040
LVEDD	0.567	−0.053
PAPs	0.114	0.145
E/e’	0.029	0.177

LVEF—left ventricle ejection fraction; LVEDD—left ventricular end-diastolic diameter; E/e’—transmitral early diastolic filling velocity/early diastolic LV myocardial velocity; PAPs—pulmonary artery systolic pressure.

**Table 5:** Correlations between evaluated biomarkers and some parameters of severity in acute HF.

Prognostic Parameter	Syndecan-1		NT-proBNP		hs-Troponin	
	*p*	r	*p*	r	*p*	r
In-hospital mortality	0.185	0.129	0.093	0.154	0.071	0.166
Total mortality at 1 month	0.097	0.149	0.015	0.222	0.087	0.157
Need for inotropic support	0.024	0.206	0.461	0.064	0.989	0.001
Non-invasive ventilation (CPAP)	0.033	0.189	0.271	0.101	0.787	0.031
Invasive ventilation (OTI)	0.182	0.127	0.964	0.004	0.796	0.024
Hospitalization duration	0.205	0.106	0.545	0.049	0.307	−0.094

CPAP—continuous positive air pressure; OTI—orotracheal intubation.

**Table 6:** Synergistic effect of syndecan-1 and classical biomarkers in mortality prediction.

Biomarkers		Syndecan-1 < 121 ng/L	Syndecan-1 > 121 ng/L	*p*-Value
	Mortality Rate (%)			
NT-proBNP < 5440 pg/mL		11.5%	23.1%	<0.01
NT-proBNP > 5440 pg/mL		15.4%	50%	<0.01
Troponin < 40 ng/L		15.4%	23.1%	0.04
Troponin > 40 ng/L		15.4%	46.1%	<0.01

**Table 7:** Serum syndecan-1 levels according to the hemodynamic parameters and documented comorbidities involved in the pathogenesis or decompensation of acute HF.

Variable	Syndecan-1 (ng/mL) Mean ± STD	*p*
Alcoholic Non-alcoholic	205.6 ± 203.9	0.457
	179.8 ± 143.4	
Smoker Non-smoker	202.9 ± 182.7	0.735
	191.3 ± 184.3	
Pre-existing chronic liver disease Without pre-existing chronic liver disease	209.5 ± 196.6	0.395
	180.9 ± 146.8	
Hepatic cytolysis at admission Without hepatic cytolysis at admission	222.3 ± 192.4	0.011
	149.3 ± 158.4	
Pre-existing chronic kidney disease Without pre-existing chronic kidney disease	207.9 ± 177.1	0.601
	189.7 ± 187.2	
Acute kidney injury early after admission Without acute kidney injury early after admission	213.1 ± 212.9	0.045
	156.3 ± 153.5	
Pre-existing cancer pathology Without pre-existing cancer pathology	255.2 ± 289.3	0.189
	189.4 ± 168.1	
Systolic blood pressure <90 mmHg Systolic blood pressure ≥90 mmHg	237.5 ± 203.1	0.036
	159.9 ± 137.7	
Heart rate ≥100/min Heart rate <100/min	235.9 ± 209.4	0.020
	158.6 ± 146.4	
SaO_2_ ≥90% SaO_2_ <90%	198.8 ± 188.3	0.709
	181.1 ± 150.3	
LV ejection fraction <40% LV ejection fraction ≥40%	200.6 ± 181.9	0.724
	190.3 ± 186.2	
Fasting blood glucose ≥126 mg/dL Fasting blood glucose <126 mg/dL	181.2 ± 169.4	0.486
	162.7 ± 172.4	
Pre-existing diabetes Without pre-existing diabetes	165.8 ± 119.3	0.395
	202.7 ± 192.3	
C-reactive protein ≥0.5 mg/dL C-reactive protein <0.5 mg/dL	175.4 ± 177.1	0.234
	132.9 ± 141.0	
BMI <30 kg/m^2^ BMI ≥30 kg/m^2^	236.3 ± 201.2	0.041
	180.0 ± 173.1	

BMI—body mass index; LV—left ventricle; SaO_2_—arterial oxygen saturation.

**Table 8:** Correlations between syndecan-1 at admission and the markers of organ dysfunction at discharge.

Pathologic Serum Level at Discharge	Syndecan-1 at Admission	
	*p*	r
AST	0.011	0.197
ALT	0.015	0.195
Urea	0.039	0.155
Creatinine	0.027	0.162

AST—aspartate aminotransferase; ALT—alanin aminotransferase.

**Table 9:** Correlations between syndecan-1 level and metabolic profile in patients with acute HF.

Metabolic Parameter	Syndecan-1	
	*p*	R
Total cholesterol	0.079	0.161
LDL-cholesterol	0.240	0.108
HDL-cholesterol	0.016	−0.219
Triglyceride	0.910	0.010

## Data Availability

All the data presented in this study are available within the article.
